# Reliability of live and video-based coding in netball using the NetballStats application

**DOI:** 10.1371/journal.pone.0269330

**Published:** 2022-06-21

**Authors:** Lyndell Bruce, Dan Dwyer, Aaron Fox

**Affiliations:** Centre for Sport Research, School of Exercise and Nutrition Sciences, Deakin University, Burwood, Victoria, Australia; Universita degli Studi di Milano, ITALY

## Abstract

Understanding reliability of performance analysis tools is important to ensure match to match comparisons can be undertaken with the knowledge of consistency between coding situations. There are few published studies examining the reliability of commonly used performance analysis tools. The aim of this project was to assess the inter- and intra-rater reliability of the NetballStats application and to make comparisons between live and video-based coding situations. Two ‘coders’ coded eight netball matches using the NetballStats application, coding each match live, then twice from video. Level of agreement was assessed for frequency counts across the variables coded. Results showed that intra-rater agreement was higher than inter-rater agreement and that reliability from video coding is better than from live coding. High frequency events automatically coded by the application and events that are well defined had greater levels of agreement than lower frequency events and subjectively judged events. Live coding situations underrepresent occurrence of events, particularly for high frequency events such as ‘possession’. To ensure reliability between coders, clubs should provide an extensive training program to coders with clear instructions on coding subjective events. Coaches should be aware that live coding underestimates some event types and factor this into their decision making processes.

## Introduction

A regular occurrence in many sporting events, particularly in the case of team sports is the use of notational analysis to analyse team and individual performance [1–3]. Through this process, feedback may be provided to coaches and athletes about how performance can be improved or to identify an upcoming opponent’s strengths and weaknesses. As a result, performance analysis provides a key element of a team’s preparation and evaluation of matches. To enable this process to occur, analysts record data to determine the value of performance indicators that are thought to be critical in determining the outcome of a match [4]. This typically involves one or more analysts coding a teams’ match live and/or editing the codes post match to ensure greater representation of the events that occurred.

When different coders are used within and across matches it is important to understand the similarity in the events being recorded between coders to ensure that accurate and consistent information is being provided to coaches and athletes. The importance of understanding the reliability of performance analysis tools and measures is well documented [5], however, published studies detailing reliability are lacking. Hughes et al. [6] found that out of 72 research papers, 70 percent did not report any reliability analysis of the analysis procedure. There have been a small number of studies examining the reliability of performance analysis products. Bradley et al. [7] assessed the reliability of ProZone MatchViewer, Liu et al. [8] reported on the inter-operator reliability of football match statistics from OPTA Sportsdata and Gong et al. [9] assessed validity and reliability of data from the Champdas Master Match Analysis System. All concluded that the tool they evaluated was reliable if operators had undergone appropriate training. These studies are beneficial as they allow practitioners to determine how confident they can be in the data collection tool they are using. However, there are many more performance analysis tools available, than have been assessed for reliability.

Most interfaces (i.e., code windows) on software packages or mobile device applications (‘apps’) allow users to customise the actions and events they collect information about. Therefore, assessing the reliability of code windows becomes challenging as it is unlikely two windows will be the same. However, there are an increasing number of tools which are not customisable and have pre-populated code windows that analysts use. Whilst a pre-populated window might appear to offer greater reliability than a customisable one, there may still be human error in the data entry process (e.g., allocation of the wrong position or player to an event) [10]. Understanding the intra- and inter-rater reliability of analysis tools is important so that players and coaches can be confident that the data they are basing decisions on is accurate and are not affected by which coder completed the coding [11]. Assessing reliability will also provide teams with some indication of the reliability across seasons if analysts change, and the week-to-week consistency if multiple analysts are used.

Sports performance is dynamic in nature, and this is particularly the case for team sports where multiple players are on the court or field for both teams at one. Due to this, when coding live, it can be challenging to capture all events for a match for one or both teams, especially when only one coder is coding the match, which may be the case in sports where funding is an issue or in lower levels of sport competition. Analysts often supplement their live code with a re-code post match where video footage can be paused or slowed down and add ‘missing’ events to the original live code. An understanding of differences between live coding and post-match coding (e.g., volume of events missed) will assist coaches in their in-game decision making processes due to a greater understanding of what details are unavailable from the live coding process. Where post match processing is not available (e.g., due to time or resources) this understanding of what might be missing from the analysis will assist coaches in providing feedback to athletes. Despite potential limitations with capturing all information live, coaches often still prefer some in match statistics to supplement their decision making processes in game.

Whilst research is beginning to examine the interaction between performance indicators in netball and the resultant performance outcomes [12, 13], there is little understanding of how these performance indicators are recorded and their subsequent reliability between teams, matches and competitions. NetballStats is a recent performance analysis tool that has been designed specifically for netball allowing data for technical performance indicators to be recorded. The NetballStats ‘app’ (Perana Sports, Australia) requires specific events to be coded and thus the coding process is not customisable. After certain events are coded, another event will automatically occur (e.g., after a goal is scored, the next centre pass is automatically allocated the correct team), streamlining the potentially complex process of coding events in a fast-paced sport like netball. Several teams within the Suncorp Super Netball competition and Victorian Netball League use the ‘app’ for their in-match and post-match analysis, however the reliability of the ‘app’ has not been established. Therefore, this study aimed to (1) examine the intra- and inter-rater reliability of the NetballStats ‘app’; and (2) compare outputs from live- versus video-coding practices. The findings of this study will quantify the reliability of data obtained from this and similar apps, and it will also indicate how data collected during a match, may differ from post-match video.

## Materials and methods

Two competition levels of netball were coded by two people or ‘coders’ (herein referred to as ‘C1’ and ‘C2’) on multiple occasions. Both coders had a minimum of one year of experience using the NetballStats ‘app’. An exemption from ethics was granted from [institute name] human research ethics committee. The matches coded were from the 2018 Suncorp Super Netball (SSN) competition and the 2018 Victorian Netball League (VNL) championship division. All matches in the analysis were coded live (‘live’) and post-match (‘video’). A total of eight matches, four from each competition level were coded. The home matches (rounds eight, nine, 12 and 13) of one SSN team were coded when the team played a home match to enable the coders to attend. The VNL matches were coded from rounds 11, 12, 16 and 18 to enable Championship division matches being played on the stadiums main court to be used. Each coder was present and coded each game live at the eight matches (four SSN and four VNL) except for C2 being unavailable due to illness for one live SSN match. Following the live matches, video of the SSN matches was obtained from the SSN club, and the video from the VNL matches was obtained from the website housing all VNL matches (https://netballvic.sportscastcloud.com/). One week post the live code, each coder then coded all matches twice from video, with at least one week separating each match code occurrence from video. The video for both competitions was filmed in line with the goal posts in an elevated position from the court surface. When coding the match live, both coders sat in an elevated position in line with the goalposts ensuring a consistent coding position across live and video coded matches.

All matches were coded using the NetballStats ‘app’ on an iPad Air. The NetballStats ‘app’ was developed specifically for coding netball matches. The NetballStats ‘app’ requires certain match parameters to be entered before a match can be coded. This includes date, home team, away team, tournament/competition, and venue. There is also the option to include team players and their playing position prior to commencing coding. As team lists were not available prior to the commencement of all matches, this information was not entered, with generic position labels (goal shooter (GS), goal attack (GA), etc) used in lieu of player names. The ‘app’ produces the same output irrespective of whether players names are attributed to positions, and it is designed to have the capacity to capture every possession in netball, including the court location of each possession. Certain events are recognised automatically, and the coder is only required to enter the player position responsible (e.g., GS, GA) and court location for these events. These events include centre pass receives, second phase, circle entries and throw ins (see Table 1 for definitions). As players in netball are restricted in the area in which they can move, the app will not allow entry of a playing position receiving a possession if they are not permitted to be in that area.

**Table 1:** List of definitions for events that are automatically recorded by the NetballStats ‘app’.

Assist name	Descriptor
Centre pass receive	Offensive player who receives first pass from the centre player on a centre pass
Second phase	Offensive player who receives next pass from the centre pass receiver
Circle entry	Offensive player who passes the ball from outside the goal circle into the goal circle, and the ball is received by a teammate
Throw-in	The ball is thrown back into play from the point where it went off the court by the team who did not touch it last

In preparation for coding, the coders indicated the direction each team was shooting prior to the commencement of the game, and which team had the first centre pass. Following this, the ‘app’ can recognise the alternating nature of centre passes in netball. Similarly, it recognises that after a quarter has been ‘locked’ (i.e., the quarter has ended and no more events will occur), that the teams change ends and shoot to the opposite end to the previous quarter. The coders were instructed to record all possessions as they occurred, inclusive of which player had the possession and where on court the possession occurred, as accurately as possible. Due to the pace of the match, during live coding this was not always possible, so coders were instructed to code as many possessions as possible, but to prioritise critical events including gains and losses, goal attempts (including success or miss), centre pass receive, second phase receive and goal circle entries. When coding from video, coders were instructed to capture all possessions in the match and be as accurate as possible. They were permitted to play and pause the video to ensure they captured all events.

Coders selected the location on the court a pass was received, then entered which playing position received the ball. When a team gains possession of the ball, the coder selects the location on court the gain was created, then clicks gain, enters the playing position and the type of gain (see Table 2 for gain options). To code a loss, the coder selects the location, then selects loss, enters the playing position and the type of loss (see Table 3 for loss options). When a goal shooter or goal attack makes an attempt at goal, the coder selects one of the four following options; goal (goal scored), miss out (the attempt misses and the ball lands out of court resulting in an opposition throw-in), GA miss rebound or GS miss rebound (selecting these two options prompts the coder to select one of the four player rebound options as the ball is still live in play–GS, GA, opposition goal defence (opp GD), or opposition goal keeper (opp GK).

**Table 2:** List of definitions for how the defending team may gain possession of the ball.

Gain possession name	Descriptor
Intercept	When a defending player gains possession by catching a pass made by an opposition player
Defensive rebound	When a goal keeper or goal defender rebound the ball after a missed shot
Opposition contact	When an offensive team player infringes the defending team player resulting in a turnover, as determined by the umpire
Tip (deflect)	When a defending team gains possession by deflecting a pass made by an opposition player, which is picked up by the defending team
Pick-up	When a player from either team picks up a ball that is loose
Forced error	When the defending team force the opposition into making an error
Opposition error	When the offensive team make an unforced error (e.g., break, held ball)
Unknown	If it is unable to be determined how the defending team gained possession

**Table 3:** List of definitions for how the offensive team may lose possession of the ball.

Lost possession name	Descriptor
Bad pass	A pass made by an offensive player that is not caught by a teammate
Dropped ball	When an offensive player does not take clean possession of the ball thrown by a teammate
Out of court	When an offensive player catches the ball or touches the ball outside the field of play
Step	When the player in possession of the ball takes more than one and half steps
Break	When an offensive player enters the centre third prior to the umpiring blowing the whistle to re-start play on a centre pass
Offside	When an attacking player enters an area of the court they are not permitted to be in
Held ball	When a player in possession of the balls holds it for greater than 3 seconds
Stopped lead	When an offensive player stops their lead prior to receiving the ball
Forced pass	When an offensive player tries to force a pass to a teammate whilst defended heavily
Replay	When a player is deemed to have possession of the ball, release it, and regain it again before another player or object (e.g., goal posts) has touched the ball
Offensive contact	When an offensive team player infringes the defending team player resulting in a turnover, as determined by the umpire
Centre Pass Infringe	When the grounded foot (first foot) of the centre player is partly outside the centre circle when the umpire blows their whistle to re-start play on a centre pass
Other	Any offensive infringement not covered

Following completion of each match code, the coders emailed the principal researcher the raw code file and related csv file. Individual CSV files across the various matches, coders and coding conditions were used in subsequent analyses.

### Data analysis

All data analysis was conducted using Python (version 3.7.6). The data from all individual CSV files was imported using the pandas package [14]. Frequency counts for events (see Table 4) coded within the NetballStats ‘app’ were determined across each individual match, coder (i.e., C1 and C2) and coding condition (i.e., Live, Video 1 and Video 2). Certain events representing a similar outcome were collapsed together (e.g., Gain inclusive of intercept, deflection, pick-up, etc) when determining frequency counts (see Table 4).

**Table 4:** List of events for which frequency counts were obtained from coded data.

Event Name	Descriptor
Attack Rebound	A rebound made by the offensive team after an unsuccessful shot on goal
Centre Pass	A pass is taken from the centre circle by the Centre of each team to recommence play after a goal has been scored or at the beginning of a quarter. The Centre Pass alternates between teams.
Centre Pass Infringe	When the grounded foot (first foot) of the C player is partly outside the centre circle when the umpire blows their whistle to re-start play on a centre pass
Centre Pass Receive	Offensive player who receives first pass from the centre player on a centre pass
Circle Entry	Offensive player who passes the ball from outside the goal circle into the goal circle, and the ball is received by a teammate
Defensive Rebound	A rebound made by the defensive team after an unsuccessful shot on goal
Gain	Occurred when the defending teams gains possession of the ball from the opposition through an intercept or deflection
Loss	Occurred when the offensive team looses possession of the ball through bad pass, force pass, offensive contact, drop ball, held ball, step, offside, break or other.
Made Goal	A successful attempt at goal by the offensive team
Missed Goal	An unsuccessful attempt at goal by the offensive team
Penalty	When a player infringed on an opposition player including contact and obstruction
Possession	When a player was in control of the ball
Second Phase	Offensive player who receives next pass from the centre pass receiver
Throw In	Offensive player who receives next pass from the centre pass receiver
Tip (Deflect)	When a defending team gains possession by deflecting a pass made by an opposition player, which is picked up by the defending team

We examined the agreement (i.e. reliability) for events identified across a series of comparisons, those being: (i) inter-rater reliability between coders across four VNL and three SSN (seven matches) from both competitions coded live; (ii) inter-rater reliability between coders across four VNL and four SSN (eight matches) from both competitions coded from video; and (iii) intra-rater reliability for both coders across four VNL and four SSN (eight matches) from both competitions coded across two video coding sessions. To assess agreement, we calculated the absolute and percentage differences (mean ± SD) between the frequency counts of the coded events across the relevant matches; along with 95% limits of agreement for the corresponding frequency counts. Level of agreement was used as it was not possible to directly correlate each possession (e.g., Kappa statistic) using timestamped data. This is due to the fast paced nature of netball (players must dispose of the ball within three seconds and often do so quicker) and the natural speed of coding that may vary between coders, particularly when coding live. Our overall goal was to understand the agreement for identifying the frequency of events as these comparisons are common in team sports rather than making statistical comparisons. The analyses undertaken provide actual values (rather than standardised values) which are more easily understood and communicated to coaches.

## Results

**Fig 1:**
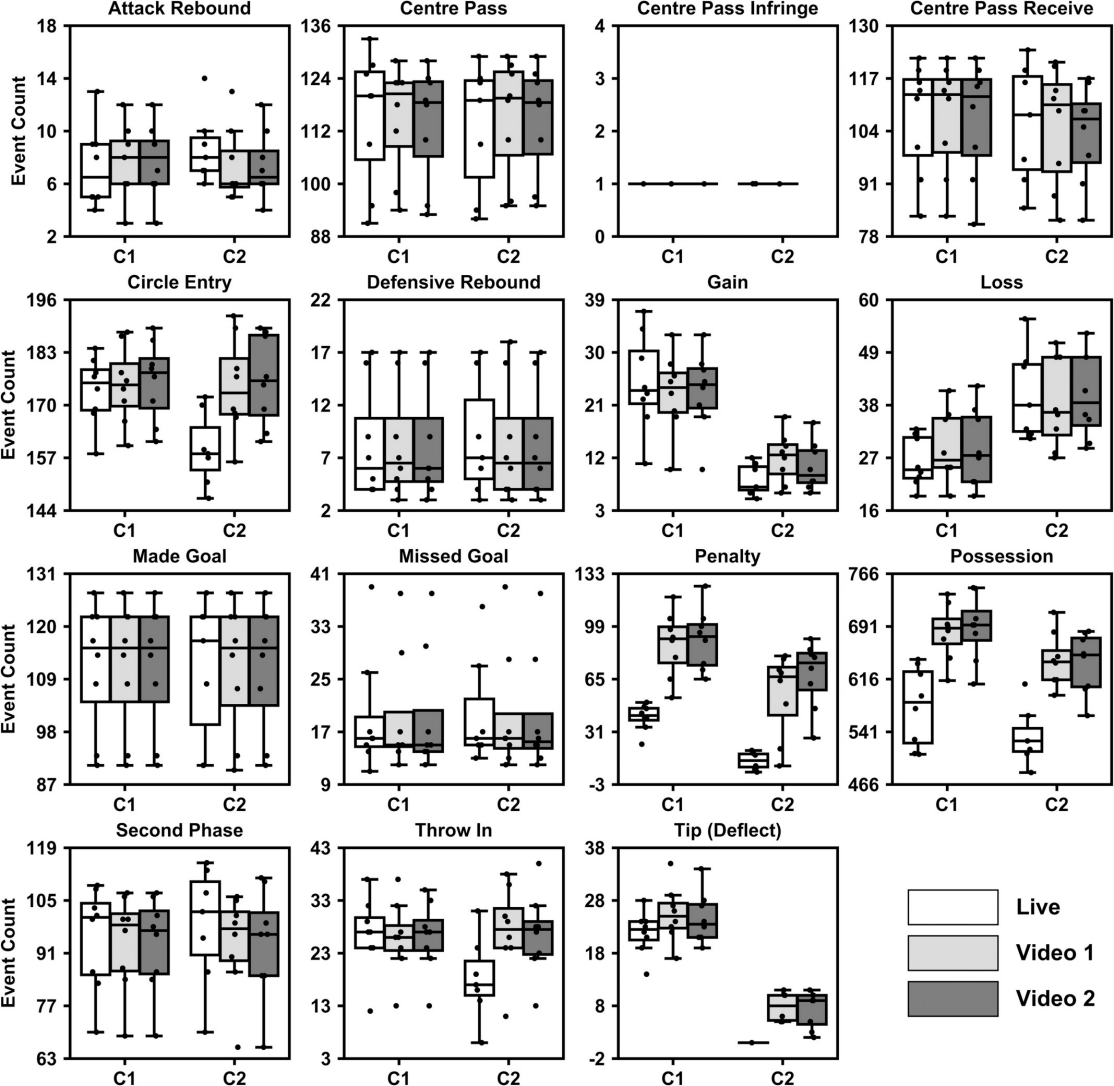
Box and whisker plot for the frequency of coded events for Victorian Netball League (VNL) and Suncorp Super Netball matches combined across the different coders (i.e., C1 and C2) and coding conditions (i.e., Live, Video 1 and Video 2). The horizontal line within boxes represents the median value, while the outer borders of the box represents the 25^th^ to 75^th^ quartiles of the data. Error bars represent the 1.5 inter-quartile range. Individual frequency counts are specified by the singular plotted points.

**Fig 2:**
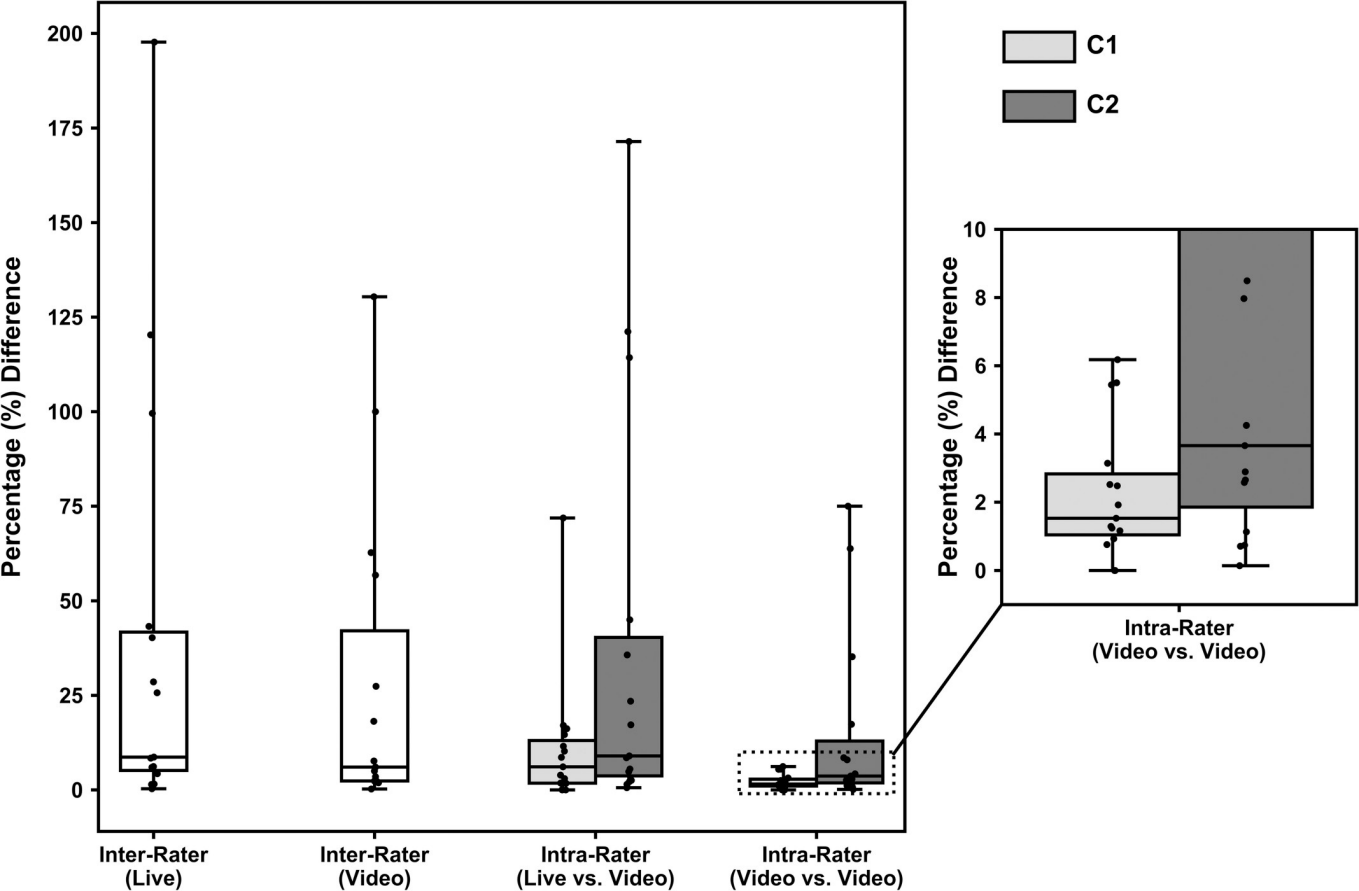
Agreement between coders and conditions. The horizontal line within boxes represents the median value, while the outer borders of the box represents the 25^th^ to 75^th^ quartiles of the data. Error bars represent the 1.5 inter-quartile range. Individual frequency counts are specified by the singular plotted points.

Frequency count data for the coded events collapsed across the different competitions for the different coders and coding conditions are displayed in Fig 1. Fig 2 shows the percentage agreement between and within coders for the different conditions (live and video). Results showed that intra-rater agreement was higher than inter-rater agreement and that reliability from video coding is better than from live coding. High frequency events automatically coded by the application and events that are well defined had greater levels of agreement than lower frequency events and subjectively judged events. Live coding situations underrepresent occurrence of events, particularly for high frequency events such as ‘possession’.

Absolute and percentage differences, and the 95% limits of agreement for the frequency of coded events are presented in S1A–S1F Table. Specifically, inter-rater reliability between coders for live-coded combined competition matches are presented in S1A Table; inter-rater reliability between coders for video-coded combined competition matches is presented in S1B Table; intra-rater reliability for C1 between live- and video-coded combined competition matches is presented in S1C Table; intra-rater reliability for C2 between live- and video-coded combined competition matches is presented in S1D Table; intra-rater reliability for C1 between two video-coding sessions for combined competition matches are presented in S1E Table; and intra-rater reliability for C2 between two video-coding sessions for combined competitions matches are presented in S1F Table.

## Discussion

This study examined the intra- and inter-rater reliability of the NetballStats ‘app’ using two coders who coded both live and post-match from video netball games. A secondary aim was to assess the difference between coding outputs when completed live versus from video. There was better agreement within versus between coders. Well defined events (e.g., centre passes) were the most consistent in all comparisons, especially for larger scale frequency events. Live coding appeared to underestimate events, compared with post-match video coding, particularly for those events with high frequencies (e.g., possessions).

Variables that were automatically captured by the app and higher in frequency (e.g., centre pass, circle entry), showed good agreement between the two coders (e.g., live centre pass, 1.43% difference, live circle entry, 8.69% difference, video centre pass, 2.20% difference, video circle entry, 2.38% difference). Furthermore, events that are well defined in netball (e.g., centre passes, centre pass received, goals (made and missed)) were the most consistently coded across both coding conditions (i.e., live and post-match video) for the larger scale frequency events. Circle entry and second phase were also relatively consistent. The combination of these events being prominent, automatically coded, and objectively well-defined are likely factors behind the consistent coding of these events. Some less common events not automatically identified by the ‘app’ also seem to be easier to identify irrespective of coder/coding condition compared to more frequent events not automatically coded (e.g., attacking rebound (*live*, 25.67% difference, *video*, 18.14% difference), defensive rebound, (*live*, 8.41% difference, *video*, 3.49% difference), throw in’s (*live*, 43.22% difference, *video*, 7.65% difference)). Again, this is likely due to the well-defined nature of the events but may also be driven by the limited number of these events in netball gameplay. The inclusion of automatically identified events within the ‘app’ is good for consistency, particularly for high frequency events. In addition, events that are well defined (e.g., throw-ins, attacking and defensive rebound) provide better consistency between coders. This provides users with confidence in using data from the ‘app’ for these events, irrespective of who completes the coding.

When comparing the two coders during live matches, variables that required a subjective judgement (e.g., gain, loss) and variables that were not automatically coded by the ‘app’ (e.g., tip, penalty) resulted in the largest differences between the coders. For example, C2 underreported gain and tip/deflect variables compared to C1, whereas they over reported losses. Given gains and losses are essentially the inverse of each other (i.e., one team’s loss would be the other team’s gain). It appears that the general event (change of possession) is being recognised but coded differently between coders. Misclassification of an event may lead to a player’s value to the team being over and underestimated if based purely on their statistical worth. However, it is unlikely that a coach would reply purely on statistics to judge a player’s contribution to a match.

For both coders, live coding tended to cause an under representation of events, particularly those with a high frequency (e.g., possession, penalty) that were not automatically identified by the ‘app’. This is not unexpected given the instructions asked coders to focus on key events. It is likely they prioritised these events over coding every single possession and all penalties that occurred. Furthermore, during live coding, due to the number of buttons to clicked and the need to look between the screen and court, it can be difficult to capture all possessions, especially after a turnover. Therefore, is not surprising these two variables (possession, penalty) were underrepresented in live coding. There were some discrepancies between the two coders in terms of what events were seemingly ‘sacrificed’ when coding live in comparison to the video code. The frequency of circle entries and throw ins was lower for C2 in the live code, whereas they were not for C1. Coder 1, even though they had fewer instances of some frequent variables (i.e., circle entries and possessions) in the live coding, they were consistent between coding sessions for all other variables. When reviewing the data, coaches and analysts should expect a lower than actual count for events that are not automatically recorded by the ‘app’ (e.g., penalties and possessions) during a live coding session than from video.

Viewing the two video coded sessions, the agreement within each coder was greater than the agreement between the coders (see Fig 2). Coder 1 was the more consistent coder between the two video coding opportunities and showed fewer absolute differences and smaller percentage differences. Penalties was the greatest difference between the two coding instances (for C1), and this only differed by an absolute value of three (6% difference). There were greater absolute differences for possessions (C1 = eight), but given the high frequency count, this percentage difference between video codes was low. Coder 2 was more inconsistent and had discrepancies between possessions, penalties, and circle entries for the two coding attempts. A simplistic solution to ensure greater consistency is to have one coder code all matches, however, this is often impractical. If multiple coders are coding for one team, any differences between the coder are important to note, as coaches may use output (i.e., statistics) from the analysis process to base decisions about player selection. If there are large discrepancies between coders, this may impact the coaching decisions. Teams should examine the consistency between their coders in both live and video-based coding instances. Teams may like to determine an error range for their coders, upon which values outside this range could be considered a true difference between matches. The values provided in this paper may provide a guide for teams on suitable error margins.

A potential limitation of this study was the use of coders with at least one year of experience. To reduce the level of subjectivity, users of NetballStats should undertake significant training to assist with making these subjective calls. If a team has multiple staff who would be coding matches, it would be important that they undertake the same training to understand the club’s operational process and definitions. It is unlikely that the subjectivity will not be fully removed given the difficulty in discriminating between, for example, a bad pass and an intercept. Even in instances where clear operational definitions have been provided, there are still limitations in the reliability of coded data [15]. In addition to training, instructions need to be clear for subjective events to remove these inconsistencies between coders, as there were smaller inconsistencies within live and video code attempts by the same coder. It has been suggested by O’Donoghue [4] that training of coders should involve discussing definitions with sample video demonstrating the definition in action. Teams should be aware that if they have multiple coders for their season, there may be more inconsistencies between data, especially for subjective events.

In summary, key events that are automatically captured by the app were relatively well captured during live performance. There were greater discrepancies between coders for events that were more subjective in nature, and it is recommended that significant training be undertaken for users of NetballStats, and clear guidelines be given around subjective events (e.g., gains, losses). Live coding resulted in fewer events being captured, particularly for high frequency events such as possessions. Coaches need to be aware of the limitations from live coding and consider this when making their decisions. Intra-rater reliability was more consistent than inter-rater reliability when using the NetballStats ‘app’. If different coders are being used by the one club, it is important to understand differences in their coding output, so coaches’ decisions are not different based purely on the coder.

## Supporting information

S1 Table Summary inter-rater reliability statistics for a) live-coded Victorian Netball League (VNL) and Suncorp Super Netball (SSN) matches combined; b) video-coded Victorian Netball League (VNL) and Suncorp Super Netball (SSN) matches combined; c) C1 live- versus video-coded Victorian Netball League (VNL) and Suncorp Super Netball (SSN) matches combined; d) C2 live- versus video-coded Victorian Netball League (VNL) and Suncorp Super Netball (SSN) matches combined; e) C1 repeat video-coding of Victorian Netball League (VNL) and Suncorp Super Netball (SSN) matches combined; f) C2 repeat video-coding of Victorian Netball League (VNL) and Suncorp Super Netball (SSN) matches combined.
(DOCX)

S1 Fig Bland Altman for inter-rater reliability statistics for live-coded Victorian Netball League (VNL) and Suncorp Super Netball (SSN) matches combined. (TIF)

S2 Fig Bland Altman for inter-rater reliability statistics for video-coded Victorian Netball League (VNL) and Suncorp Super Netball (SSN) matches combined. (TIF)

S3 Fig Bland Altman for intra-rater reliability statistics for C1 live- versus video-coded Victorian Netball League (VNL) and Suncorp Super Netball (SSN) matches combined. (TIF)

S4 Fig Bland Altman for intra-rater reliability statistics for C2 live- versus video-coded Victorian Netball League (VNL) and Suncorp Super Netball (SSN) matches combined. (TIF)

S5 Fig Bland Altman for intra-rater reliability statistics for C1 repeat video-coding of Victorian Netball League (VNL) and Suncorp Super Netball (SSN) matches combined. (TIF)

S6 Fig Bland Altman for intra-rater reliability statistics for C2 repeat video-coding of Victorian Netball League (VNL) and Suncorp Super Netball (SSN) matches combined. (TIF)

S1 Dataset (CSV)
