# Supplementary material for: Reliability of live and video-based coding in netball using the NetballStats application
Source: PLoS One. 2022 Jun 21;17(6):e0269330. doi: 10.1371/journal.pone.0269330 (PMC9212167; doi:10.1371/journal.pone.0269330)
Supplement: S1 Table — Summary inter-rater reliability statistics for a) live-coded Victorian Netball League (VNL) and Suncorp Super Netball (SSN) matches combined; b) video-coded Victorian Netball League (VNL) and Suncorp Super Netball (SSN) matches combined; c) C1 live- versus video-coded Victorian Netball League (VNL) and Suncorp Super Netball (SSN) matches combined; d) C2 live- versus video-coded Victorian Netball League (VNL) and Suncorp Super Netball (SSN) matches combined; e) C1 repeat video-coding of Victorian Netball League (VNL) and Suncorp Super Netball (SSN) matches combined; f) C2 repeat video-coding of Victorian Netball League (VNL) and Suncorp Super Netball (SSN) matches combined. (DOCX) [file pone.0269330.s001.docx]

**Supplementary Information**

Supplementary Table 1a. Summary inter-rater reliability statistics for live-coded Victorian Netball League (VNL) and Suncorp Super Netball (SSN) matches combined

|  | **Mean (± SD) Coded No. of Events** | | **Absolute Difference (± SD)** | **Percentage Difference (± SD)** | **Limits of Agreement** | | **ICC** |
| --- | --- | --- | --- | --- | --- | --- | --- |
|  | **C1 Live** | **C2 Live** |  |  | **Lower** | **Upper** |  |
| Attack Rebound | 7.00 ± 2.98 | 8.71 ± 2.49 | 1.71 ± 1.48 | 25.67 ± 23.97 | -1.20 | 4.62 | 0.921* |
| Centre Pass | 114.29 ± 15.13 | 112.86 ± 13.79 | 1.71 ± 1.48 | 1.43 ± 1.12 | -4.88 | 2.02 | 0.996* |
| Centre Pass Infringe | 0.00 ± 0.00 | 0.14 ± 0.35 | 0.14 ± 0.35 | 28.57 ± 69.99 | -0.54 | 0.83 | N/A |
| Centre Pass Receive | 106.29 ± 13.74 | 105.86 ± 13.71 | 1.57 ± 1.50 | 1.53 ± 1.44 | -4.60 | 3.74 | 0.994* |
| Circle Entry | 173.57 ± 8.36 | 159.14 ± 8.48 | 14.43 ± 4.98 | 8.69 ± 2.97 | -24.19 | -4.66 | 0.904* |
| Defensive Rebound | 8.86 ± 5.11 | 8.86 ± 5.17 | 0.57 ± 0.49 | 8.41 ± 10.18 | -1.48 | 1.48 | 0.995* |
| Gain ^a^ | 25.71 ± 7.99 | 8.29 ± 2.49 | 17.43 ± 6.61 | 99.57 ± 25.96 | -30.38 | -4.48 | 0.548 |
| Loss ^b^ | 26.71 ± 4.92 | 40.43 ± 8.76 | 13.71 ± 4.98 | 40.26 ± 10.43 | 3.96 | 23.47 | 0.860* |
| Made Goal | 111.43 ± 13.45 | 111.14 ± 13.85 | 0.29 ± 0.70 | 0.31 ± 0.76 | -1.66 | 1.09 | 0.999* |
| Missed Goal | 19.86 ± 8.90 | 19.86 ± 7.83 | 1.14 ± 0.99 | 5.97 ± 5.29 | -2.96 | 2.96 | 0.992* |
| Penalty ^c^ | 39.57 ± 8.30 | 10.57 ± 6.48 | 29.00 ± 8.42 | 120.30 ± 41.04 | -45.50 | -12.50 | 0.620 |
| Possession | 568.86 ± 50.28 | 534.00 ± 37.98 | 34.86 ± 26.11 | 6.17 ± 4.61 | -86.04 | 16.32 | 0.906* |
| Second Phase | 94.29 ± 13.69 | 98.29 ± 14.87 | 4.29 ± 3.01 | 4.30 ± 3.13 | -2.63 | 10.63 | 0.986* |
| Throw In | 26.86 ± 7.20 | 18.14 ± 7.28 | 8.71 ± 3.28 | 43.22 ± 19.23 | -15.15 | -2.28 | 0.946* |
| Tip (Deflect) | 21.57 ± 4.03 | 0.14 ± 0.35 | 21.43 ± 3.96 | 197.71 ± 5.60 | -29.19 | -13.67 | N/A |
| a – combined total for all ‘gain’ variables; b – combined total for all ‘loss’ variables; c – combined total for all ‘penalty’ variables; C1 – coder 1; C2 – coder 2; SD – standard deviation; ICC – intra-class correlation coefficient (* indicative of statistically significant ICC of p < 0.05); N/A – not assessable | | | | | | | |

Supplementary Table 1b. Summary inter-rater reliability statistics for video-coded Victorian Netball League (VNL) and Suncorp Super Netball (SSN) matches combined

|  | **Mean (± SD) Coded No. of Events** | | **Absolute Difference (± SD)** | **Percentage Difference (± SD)** | **Limits of Agreement** | | **ICC** |
| --- | --- | --- | --- | --- | --- | --- | --- |
|  | **C1 Video** | **C2 Video** |  |  | **Lower** | **Upper** |  |
| Attack Rebound | 7.75 ± 2.59 | 7.38 ± 2.64 | 1.12 ± 0.78 | 18.14 ± 15.95 | -2.96 | 2.21 | 0.932* |
| Centre Pass | 114.88 ± 11.77 | 115.12 ± 12.58 | 2.50 ± 1.00 | 2.20 ± 0.86 | -5.00 | 5.50 | 0.988* |
| Centre Pass Infringe | 0.12 ± 0.33 | 0.38 ± 0.48 | 0.50 ± 0.50 | 100.00 ± 100.00 | -1.05 | 1.55 | N/A |
| Centre Pass Receive | 107.38 ± 13.04 | 105.25 ± 13.81 | 2.38 ± 1.41 | 2.33 ± 1.52 | -5.58 | 1.33 | 0.996* |
| Circle Entry | 175.00 ± 8.99 | 174.62 ± 11.26 | 4.12 ± 2.47 | 2.38 ± 1.43 | -9.77 | 9.02 | 0.941* |
| Defensive Rebound | 8.38 ± 5.00 | 8.38 ± 5.31 | 0.25 ± 0.43 | 3.49 ± 7.32 | -0.98 | 0.98 | 0.998* |
| Gain ^a^ | 23.00 ± 6.44 | 12.00 ± 4.00 | 11.00 ± 4.06 | 62.73 ± 22.75 | -18.96 | -3.04 | 0.832* |
| Loss ^b^ | 29.25 ± 6.87 | 38.50 ± 8.79 | 9.25 ± 3.03 | 27.38 ± 7.87 | 3.31 | 15.19 | 0.962* |
| Made Goal | 111.75 ± 12.61 | 111.50 ± 12.85 | 0.25 ± 0.43 | 0.25 ± 0.44 | -1.10 | 0.60 | 1.000* |
| Missed Goal | 19.38 ± 8.56 | 19.50 ± 8.67 | 0.88 ± 0.78 | 5.02 ± 5.49 | -2.16 | 2.41 | 0.995* |
| Penalty ^c^ | 87.25 ± 19.65 | 55.00 ± 25.16 | 32.25 ± 11.96 | 56.79 ± 41.35 | -55.68 | -8.82 | 0.925* |
| Possession | 683.25 ± 37.28 | 643.25 ± 35.94 | 40.00 ± 22.03 | 6.03 ± 3.27 | -83.19 | 3.19 | 0.900* |
| Second Phase | 93.75 ± 12.08 | 93.62 ± 12.28 | 1.62 ± 0.86 | 1.89 ± 1.28 | -3.72 | 3.47 | 0.994* |
| Throw In | 25.88 ± 6.58 | 27.25 ± 7.82 | 1.88 ± 1.36 | 7.65 ± 5.70 | -2.28 | 5.03 | 0.983* |
| Tip (Deflect) | 25.38 ± 4.97 | 5.88 ± 4.04 | 19.50 ± 4.77 | 130.39 ± 42.44 | -28.85 | -10.15 | 0.731 |
| a – combined total for all ‘gain’ variables; b – combined total for all ‘loss’ variables; c – combined total for all ‘penalty’ variables; C1 – coder 1; C2 – coder 2; SD – standard deviation; ICC – intra-class correlation coefficient (* indicative of statistically significant ICC of p < 0.05); N/A – not assessable | | | | | | | |

Supplementary Table 1c. Summary intra-rater reliability statistics for C1 live- versus video-coded Victorian Netball League (VNL) and Suncorp Super Netball (SSN) matches combined

|  | **Mean (± SD) Coded No. of Events** | | **Absolute Difference (± SD)** | **Percentage Difference (± SD)** | **Limits of Agreement** | | **ICC** |
| --- | --- | --- | --- | --- | --- | --- | --- |
|  | **C1 Live** | **C1 Video** |  |  | **Lower** | **Upper** |  |
| Attack Rebound | 7.25 ± 2.86 | 7.75 ± 2.59 | 1.00 ± 0.87 | 16.20 ± 14.50 | -1.90 | 2.90 | 0.947* |
| Centre Pass | 115.00 ± 14.27 | 114.88 ± 11.77 | 3.38 ± 1.80 | 3.00 ± 1.87 | -7.62 | 7.37 | 0.978* |
| Centre Pass Infringe | 0.12 ± 0.33 | 0.12 ± 0.33 | 0.00 ± 0.00 | 0.00 ± 0.00 | 0.00 | 0.00 | N/A |
| Centre Pass Receive | 107.25 ± 13.10 | 107.38 ± 13.04 | 0.12 ± 0.33 | 0.12 ± 0.33 | -0.52 | 0.77 | 1.000* |
| Circle Entry | 173.62 ± 7.83 | 175.00 ± 8.99 | 3.12 ± 3.06 | 1.76 ± 1.67 | -6.76 | 9.51 | 0.935* |
| Defensive Rebound | 8.25 ± 5.04 | 8.38 ± 5.00 | 0.38 ± 0.48 | 8.62 ± 11.43 | -1.05 | 1.30 | 0.996* |
| Gain ^a^ | 24.88 ± 7.80 | 23.00 ± 6.44 | 2.88 ± 1.83 | 11.55 ± 6.17 | -7.46 | 3.71 | 0.959* |
| Loss ^b^ | 26.12 ± 4.86 | 29.25 ± 6.87 | 3.12 ± 2.62 | 10.24 ± 7.87 | -2.01 | 8.26 | 0.949* |
| Made Goal | 111.75 ± 12.61 | 111.75 ± 12.61 | 0.00 ± 0.00 | 0.00 ± 0.00 | 0.00 | 0.00 | 1.000* |
| Missed Goal | 19.25 ± 8.48 | 19.38 ± 8.56 | 1.12 ± 0.78 | 6.11 ± 3.18 | -2.55 | 2.80 | 0.994* |
| Penalty ^c^ | 40.38 ± 8.05 | 87.25 ± 19.65 | 46.88 ± 18.62 | 71.90 ± 24.02 | 10.37 | 83.38 | 0.374 |
| Possession | 577.50 ± 52.30 | 683.25 ± 37.28 | 105.75 ± 27.76 | 17.03 ± 5.19 | 51.34 | 160.16 | 0.897* |
| Second Phase | 95.00 ± 12.94 | 93.75 ± 12.08 | 1.75 ± 1.20 | 1.81 ± 1.15 | -4.61 | 2.11 | 0.995* |
| Throw In | 26.50 ± 6.80 | 25.88 ± 6.58 | 0.88 ± 0.78 | 3.92 ± 3.46 | -2.57 | 1.32 | 0.994* |
| Tip (Deflect) | 21.88 ± 3.85 | 25.38 ± 4.97 | 3.50 ± 2.12 | 14.60 ± 7.76 | -0.66 | 7.66 | 0.940* |
| a – combined total for all ‘gain’ variables; b – combined total for all ‘loss’ variables; c – combined total for all ‘penalty’ variables; C1 – coder 1; SD – standard deviation; ICC – intra-class correlation coefficient (* indicative of statistically significant ICC of p < 0.05); N/A – not assessable | | | | | | | |

Supplementary Table 1d. Summary intra-rater reliability statistics for C2 live- versus video-coded Victorian Netball League (VNL) and Suncorp Super Netball (SSN) matches combined

|  | **Mean (± SD) Coded No. of Events** | | **Absolute Difference (± SD)** | **Percentage Difference (± SD)** | **Limits of Agreement** | | **ICC** |
| --- | --- | --- | --- | --- | --- | --- | --- |
|  | **C2 Live** | **C2 Video** |  |  | **Lower** | **Upper** |  |
| Attack Rebound | 8.71 ± 2.49 | 7.00 ± 2.62 | 1.71 ± 1.16 | 23.44 ± 17.46 | -3.99 | 0.56 | 0.946* |
| Centre Pass | 112.86 ± 13.79 | 114.43 ± 13.31 | 1.57 ± 1.40 | 1.46 ± 1.28 | -1.17 | 4.31 | 0.997* |
| Centre Pass Infringe | 0.14 ± 0.35 | 0.43 ± 0.49 | 0.57 ± 0.49 | 114.29 ± 98.97 | -1.09 | 1.66 | N/A |
| Centre Pass Receive | 105.86 ± 13.71 | 104.29 ± 14.51 | 2.14 ± 1.12 | 2.15 ± 1.31 | -5.18 | 2.04 | 0.996* |
| Circle Entry | 159.14 ± 8.48 | 174.29 ± 12.00 | 15.14 ± 4.97 | 8.99 ± 2.64 | 5.40 | 24.88 | 0.939* |
| Defensive Rebound | 8.86 ± 5.17 | 9.00 ± 5.40 | 0.43 ± 0.73 | 2.55 ± 4.31 | -1.49 | 1.78 | 0.994* |
| Gain ^a^ | 8.29 ± 2.49 | 12.29 ± 4.20 | 4.00 ± 3.34 | 35.68 ± 31.16 | -2.54 | 10.54 | 0.695 |
| Loss ^b^ | 40.43 ± 8.76 | 39.29 ± 9.13 | 3.14 ± 1.46 | 8.52 ± 4.66 | -7.55 | 5.27 | 0.965* |
| Made Goal | 111.14 ± 13.85 | 111.14 ± 13.71 | 0.57 ± 0.73 | 0.60 ± 0.78 | -1.81 | 1.81 | 0.999* |
| Missed Goal | 19.86 ± 7.83 | 20.00 ± 9.17 | 1.00 ± 1.07 | 4.85 ± 5.08 | -2.71 | 3.00 | 0.993* |
| Penalty ^c^ | 10.57 ± 6.48 | 53.00 ± 26.30 | 42.43 ± 24.00 | 121.15 ± 43.14 | -4.61 | 89.47 | 0.501 |
| Possession | 534.00 ± 37.98 | 633.57 ± 26.96 | 99.57 ± 33.92 | 17.20 ± 5.89 | 33.10 | 166.05 | 0.639 |
| Second Phase | 98.29 ± 14.87 | 92.86 ± 12.94 | 5.43 ± 3.89 | 5.51 ± 3.64 | -13.05 | 2.19 | 0.980* |
| Throw In | 18.14 ± 7.28 | 27.71 ± 8.26 | 9.57 ± 3.81 | 44.98 ± 17.43 | 2.10 | 17.04 | 0.936* |
| Tip (Deflect) | 0.14 ± 0.35 | 5.29 ± 3.99 | 5.43 ± 3.81 | 171.43 ± 69.99 | -3.07 | 13.35 | N/A |
| a – combined total for all ‘gain’ variables; b – combined total for all ‘loss’ variables; c – combined total for all ‘penalty’ variables; C2 – coder 2; SD – standard deviation; ICC – intra-class correlation coefficient (* indicative of statistically significant ICC of p < 0.05); N/A – not assessable | | | | | | | |

Supplementary Table 1e. Summary intra-rater reliability statistics for C1 repeat video-coding of Victorian Netball League (VNL) and Suncorp Super Netball (SSN) matches combined

|  | **Mean (± SD) Coded No. of Events** | | **Absolute Difference (± SD)** | **Percentage Difference (± SD)** | **Limits of Agreement** | | **ICC** |
| --- | --- | --- | --- | --- | --- | --- | --- |
|  | **C1 Video 1** | **C1 Video 2** |  |  | **Lower** | **Upper** |  |
| Attack Rebound | 7.75 ± 2.59 | 7.75 ± 2.63 | 0.25 ± 0.43 | 3.14 ± 5.45 | -0.98 | 0.98 | 0.991* |
| Centre Pass | 114.88 ± 11.77 | 113.75 ± 12.43 | 1.62 ± 1.80 | 1.53 ± 1.76 | -5.33 | 3.08 | 0.992* |
| Centre Pass Infringe | 0.12 ± 0.33 | 0.12 ± 0.33 | 0.00 ± 0.00 | 0.00 ± 0.00 | 0.00 | 0.00 | N/A |
| Centre Pass Receive | 107.38 ± 13.04 | 106.88 ± 13.57 | 0.75 ± 0.83 | 0.76 ± 0.89 | -2.46 | 1.46 | 0.999* |
| Circle Entry | 175.00 ± 8.99 | 175.88 ± 9.28 | 1.62 ± 0.99 | 0.93 ± 0.57 | -2.44 | 4.19 | 0.991* |
| Defensive Rebound | 8.38 ± 5.00 | 8.25 ± 5.04 | 0.12 ± 0.33 | 1.92 ± 5.09 | -0.77 | 0.52 | 0.999* |
| Gain ^a^ | 23.00 ± 6.44 | 23.38 ± 6.46 | 0.62 ± 0.70 | 2.52 ± 2.72 | -1.30 | 2.05 | 0.996* |
| Loss ^b^ | 29.25 ± 6.87 | 29.00 ± 7.68 | 1.50 ± 1.12 | 5.50 ± 4.84 | -3.88 | 3.38 | 0.984* |
| Made Goal | 111.75 ± 12.61 | 111.75 ± 12.61 | 0.00 ± 0.00 | 0.00 ± 0.00 | 0.00 | 0.00 | 1.000* |
| Missed Goal | 19.38 ± 8.56 | 19.38 ± 8.77 | 0.25 ± 0.43 | 1.29 ± 2.39 | -0.98 | 0.98 | 0.999* |
| Penalty ^c^ | 87.25 ± 19.65 | 90.50 ± 18.49 | 4.75 ± 5.72 | 6.18 ± 9.06 | -9.85 | 16.35 | 0.968* |
| Possession | 683.25 ± 37.28 | 688.88 ± 43.67 | 8.12 ± 5.28 | 1.16 ± 0.70 | -9.84 | 21.09 | 0.990* |
| Second Phase | 93.75 ± 12.08 | 93.38 ± 12.08 | 1.12 ± 1.05 | 1.24 ± 1.21 | -3.30 | 2.55 | 0.996* |
| Throw In | 25.88 ± 6.58 | 26.12 ± 6.37 | 0.75 ± 0.83 | 2.48 ± 2.75 | -1.89 | 2.39 | 0.993* |
| Tip (Deflect) | 25.38 ± 4.97 | 24.62 ± 4.55 | 1.25 ± 0.66 | 5.44 ± 3.43 | -3.10 | 1.60 | 0.984* |
| a – combined total for all ‘gain’ variables; b – combined total for all ‘loss’ variables; c – combined total for all ‘penalty’ variables; C1 – coder 1; SD – standard deviation; ICC – intra-class correlation coefficient (* indicative of statistically significant ICC of p < 0.05); N/A – not assessable | | | | | | | |

Supplementary Table 1f. Summary intra-rater reliability statistics for C2 repeat video-coding of Victorian Netball League (VNL) and Suncorp Super Netball (SSN) matches combined

|  | **Mean (± SD) Coded No. of Events** | | **Absolute Difference (± SD)** | **Percentage Difference (± SD)** | **Limits of Agreement** | | **ICC** |
| --- | --- | --- | --- | --- | --- | --- | --- |
|  | **C2 Video 1** | **C2 Video 2** |  |  | **Lower** | **Upper** |  |
| Attack Rebound | 7.38 ± 2.64 | 7.38 ± 2.39 | 0.50 ± 0.50 | 7.97 ± 8.78 | -1.39 | 1.39 | 0.980* |
| Centre Pass | 115.12 ± 12.58 | 114.50 ± 11.90 | 0.88 ± 0.93 | 0.74 ± 0.76 | -2.80 | 1.55 | 0.998* |
| Centre Pass Infringe | 0.38 ± 0.48 | 0.00 ± 0.00 | 0.38 ± 0.48 | 75.00 ± 96.82 | -1.32 | 0.57 | N/A |
| Centre Pass Receive | 105.25 ± 13.81 | 103.38 ± 11.46 | 3.12 ± 1.54 | 2.89 ± 1.34 | -7.63 | 3.88 | 0.986* |
| Circle Entry | 174.62 ± 11.26 | 176.12 ± 10.48 | 4.50 ± 3.00 | 2.58 ± 1.69 | -8.68 | 11.68 | 0.939* |
| Defensive Rebound | 8.38 ± 5.31 | 8.25 ± 5.09 | 0.12 ± 0.33 | 0.71 ± 1.89 | -0.77 | 0.52 | 0.999* |
| Gain ^a^ | 12.00 ± 4.00 | 10.50 ± 3.87 | 1.75 ± 1.20 | 17.35 ± 11.59 | -4.44 | 1.44 | 0.962* |
| Loss ^b^ | 38.50 ± 8.79 | 40.00 ± 8.37 | 1.50 ± 1.41 | 4.25 ± 4.08 | -1.27 | 4.27 | 0.993* |
| Made Goal | 111.50 ± 12.85 | 111.62 ± 12.65 | 0.12 ± 0.33 | 0.14 ± 0.37 | -0.52 | 0.77 | 1.000* |
| Missed Goal | 19.50 ± 8.67 | 19.25 ± 8.45 | 0.25 ± 0.43 | 1.13 ± 2.18 | -1.10 | 0.60 | 0.999* |
| Penalty ^c^ | 55.00 ± 25.16 | 67.75 ± 20.28 | 14.75 ± 6.30 | 35.22 ± 32.28 | -6.32 | 31.82 | 0.953* |
| Possession | 643.25 ± 35.94 | 638.88 ± 40.80 | 23.62 ± 9.59 | 3.66 ± 1.44 | -53.61 | 44.86 | 0.881* |
| Second Phase | 93.62 ± 12.28 | 93.50 ± 13.79 | 2.62 ± 2.12 | 2.65 ± 2.11 | -6.73 | 6.48 | 0.983* |
| Throw In | 27.25 ± 7.82 | 26.62 ± 7.35 | 2.12 ± 0.78 | 8.49 ± 3.75 | -4.89 | 3.64 | 0.979* |
| Tip (Deflect) | 5.88 ± 4.04 | 7.38 ± 3.28 | 2.25 ± 2.86 | 63.82 ± 81.03 | -5.00 | 8.00 | 0.870* |
| a – combined total for all ‘gain’ variables; b – combined total for all ‘loss’ variables; c – combined total for all ‘penalty’ variables; C2 – coder 2; SD – standard deviation; ICC – intra-class correlation coefficient (* indicative of statistically significant ICC of p < 0.05); N/A – not assessable | | | | | | | |
