# Supplementary figures and images for: Reliability of live and video-based coding in netball using the NetballStats application
Source: PLoS One. 2022 Jun 21;17(6):e0269330. doi: 10.1371/journal.pone.0269330 (PMC9212167; doi:10.1371/journal.pone.0269330)

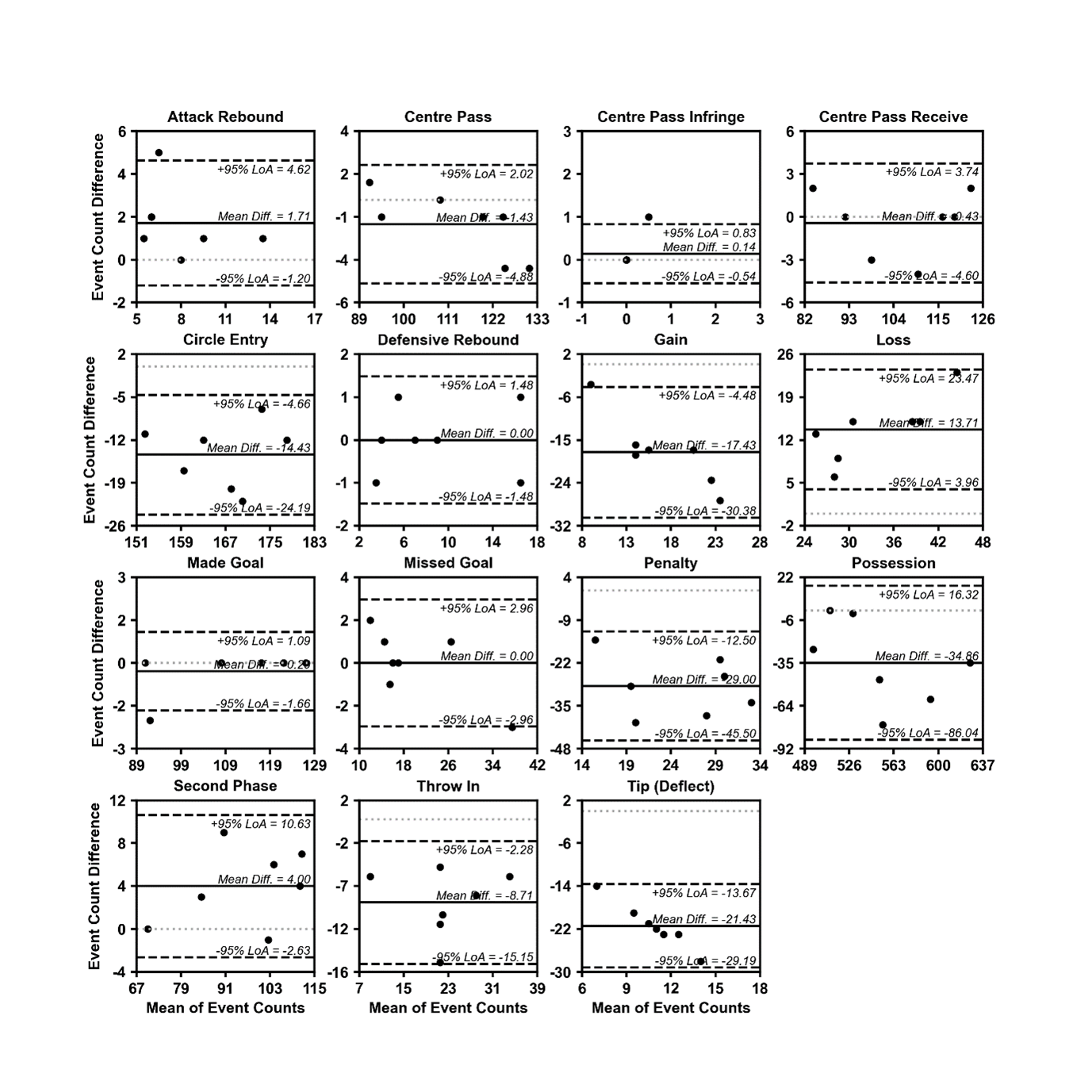

Supplement: S1 Fig — (TIF) [file pone.0269330.s002.tif]

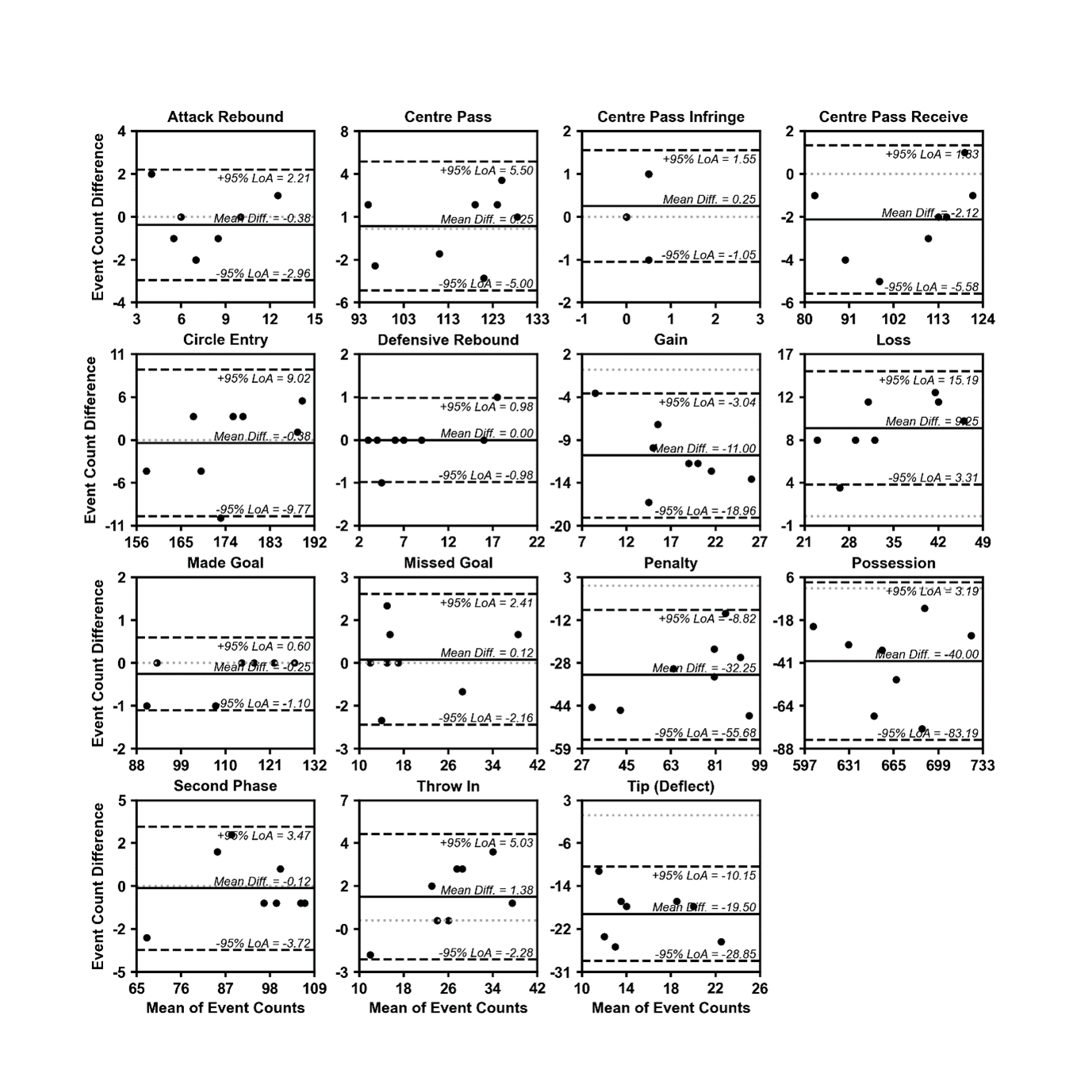

Supplement: S2 Fig — (TIF) [file pone.0269330.s003.tif]

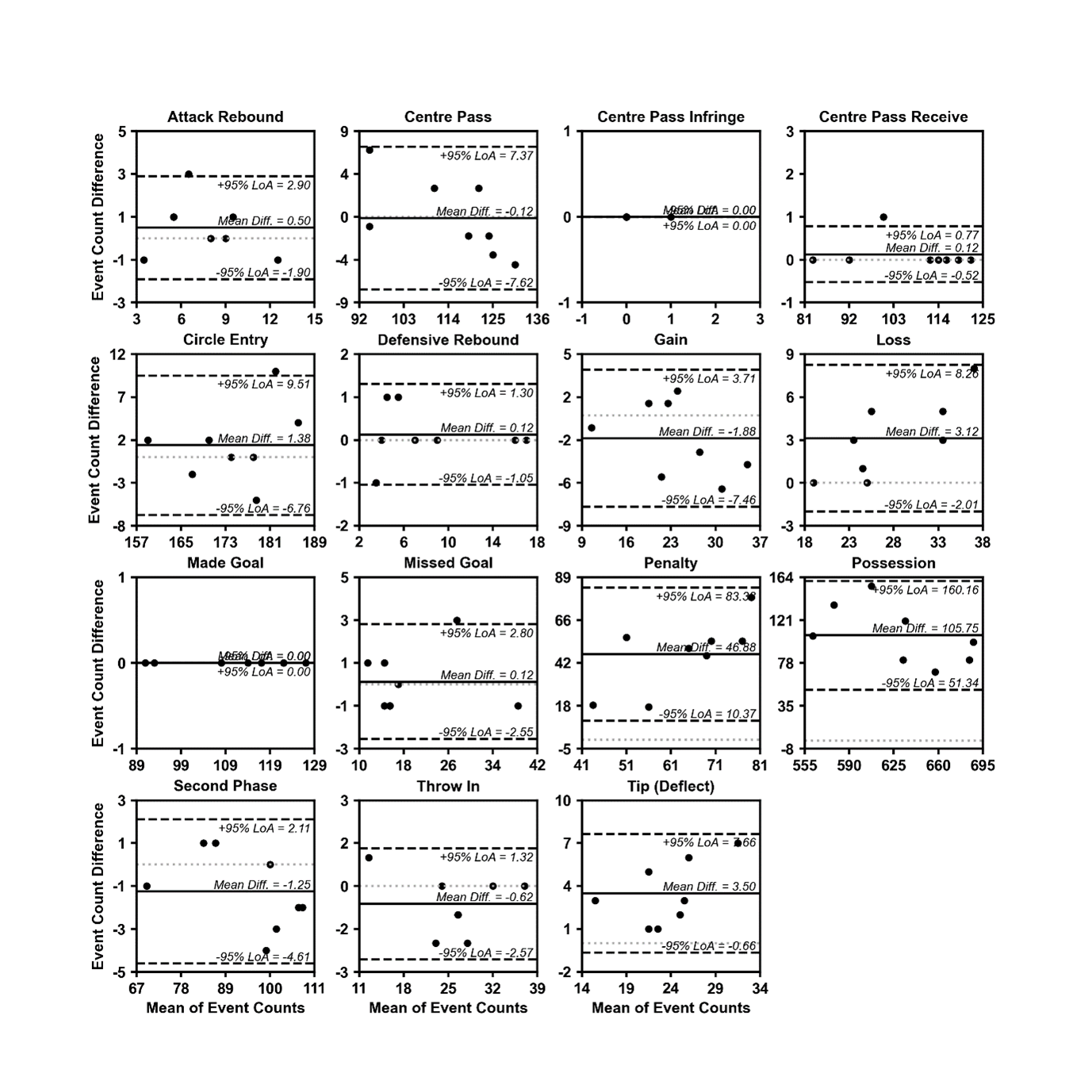

Supplement: S3 Fig — (TIF) [file pone.0269330.s004.tif]

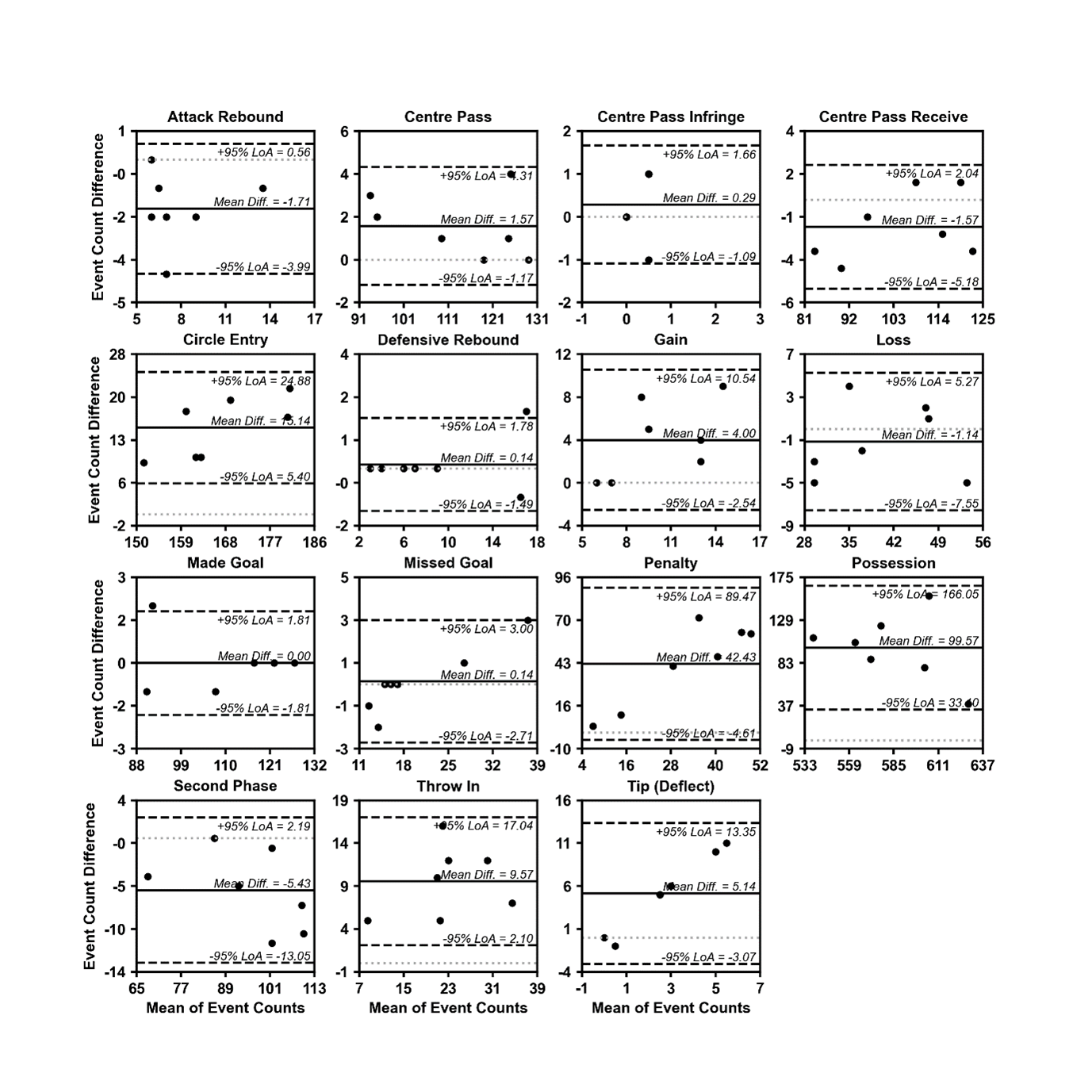

Supplement: S4 Fig — (TIF) [file pone.0269330.s005.tif]

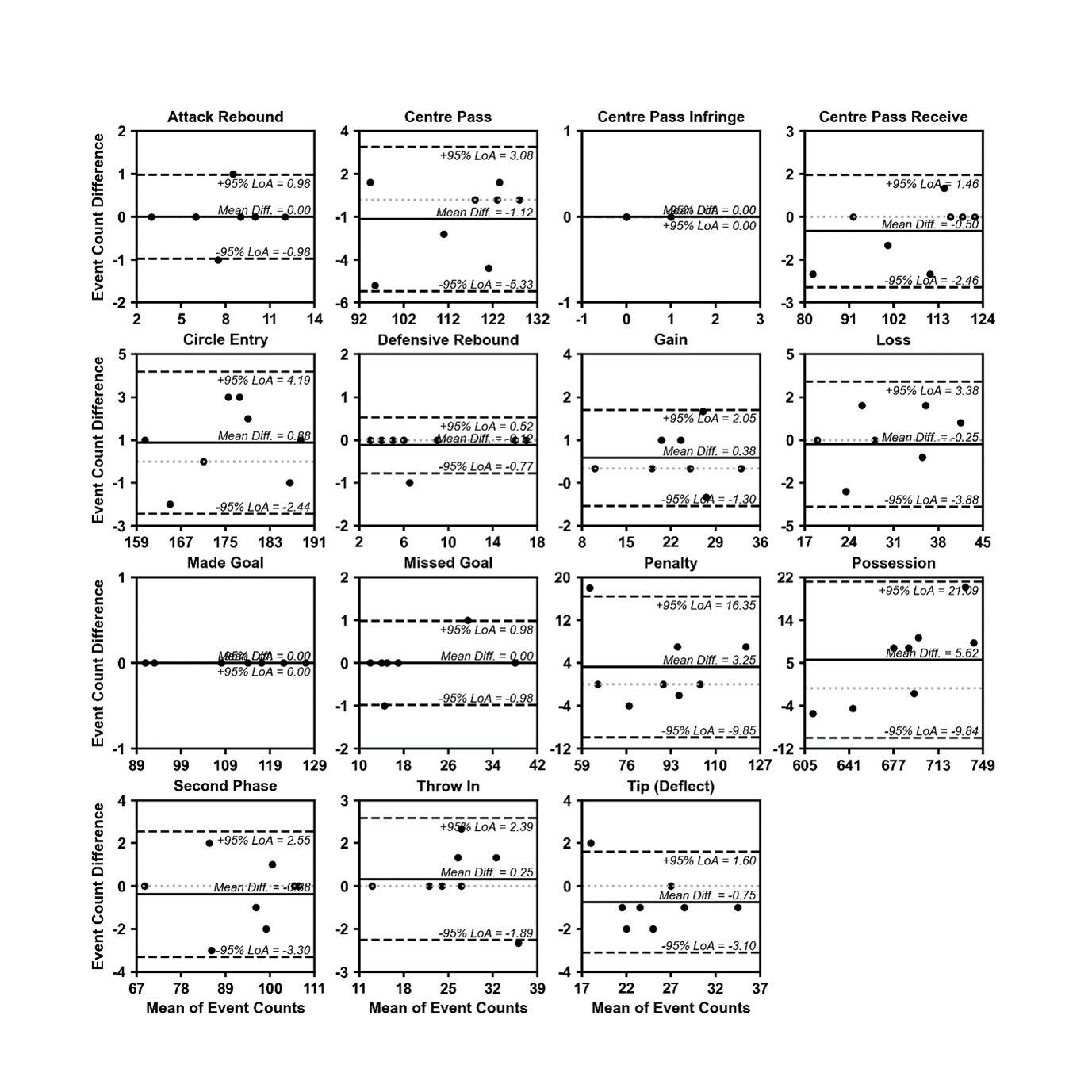

Supplement: S5 Fig — (TIF) [file pone.0269330.s006.tif]

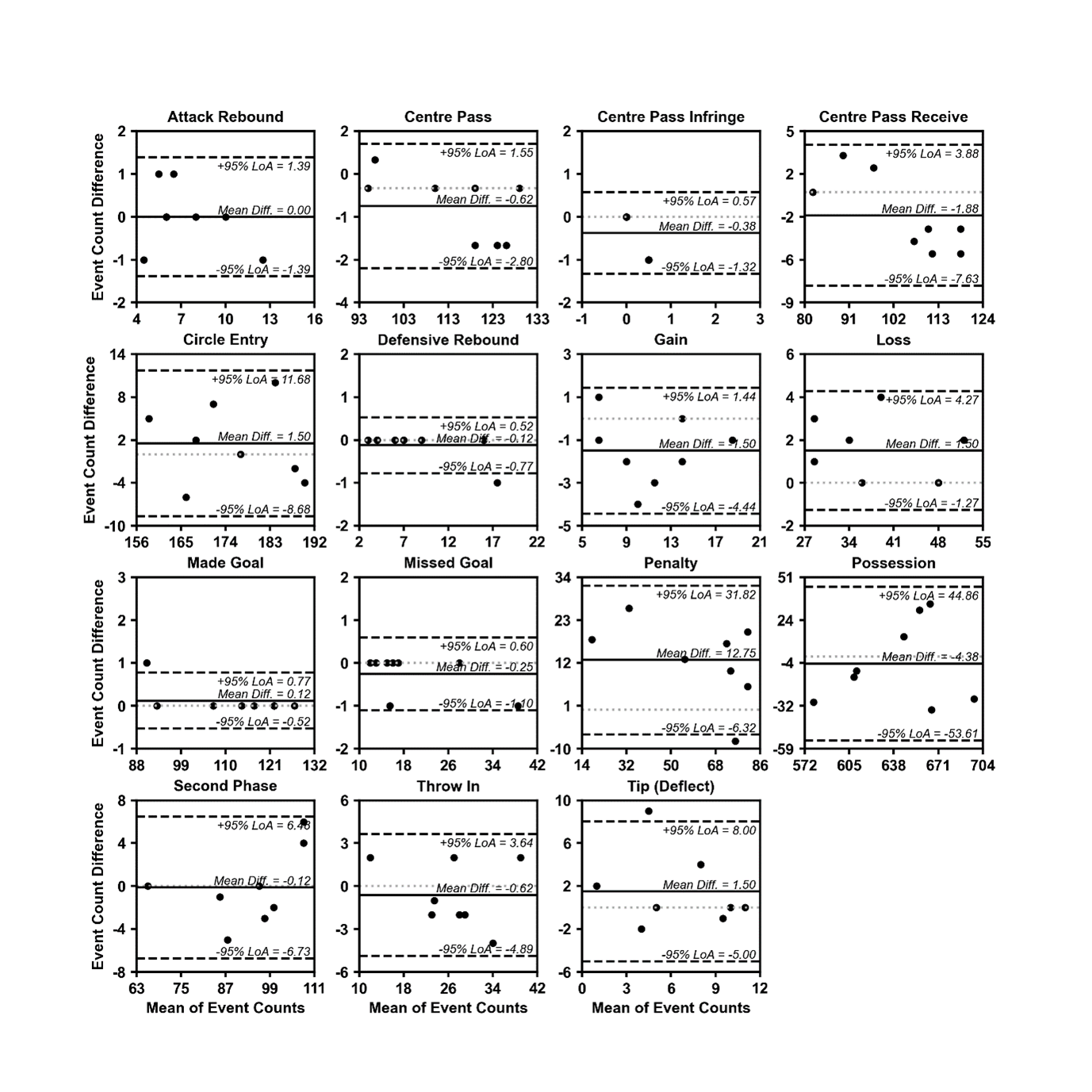

Supplement: S6 Fig — (TIF) [file pone.0269330.s007.tif]
